# Draft Genome Sequences of Three Escherichia coli Sequence Type 131 Strains

**DOI:** 10.1128/MRA.01474-18

**Published:** 2019-01-10

**Authors:** Qingwen He, Jiangqing Huang, Bin Li

**Affiliations:** a Department of Clinical Laboratory, Fujian Medical University Union Hospital, Fuzhou, Fujian, China; b Department of Clinical Laboratory, the Second Affiliated Hospital of Xiamen Medical College, Xiamen, Fujian, China; Georgia Institute of Technology

## Abstract

Escherichia coli sequence type 131 (ST131) is an important global health issue nowadays and is responsible for many clinical infections. Here, we present the complete genome sequences of two ST131 clinical isolates and one ST131 fecal isolate.

## ANNOUNCEMENT

Escherichia coli sequence type 131 (ST131) has recently emerged as an extensively antimicrobial-resistant E. coli clonal group throughout the world and is driving a rapid increase in the spread of antimicrobial resistance in E. coli (1, 2). This clonal group is an epidemiologically important clone among the extended-spectrum beta-lactamase (ESBL)-producing E. coli strains and frequently shows high resistance to fluoroquinolones (1, 2). ST131 is a highly successful E. coli clone, as strains of this clone can cause a wide variety of clinical infections. Previous studies showed that human gut flora is a possible reservoir of E. coli ST131 strains (3, 4). We describe here the complete genome sequences of three E. coli ST131 strains, including one isolated from a fecal sample from a healthy volunteer.

Three E. coli ST131 strains were isolated in 2016 as part of a screening program for gut E. coli ST131 colonization status among healthy persons and for E. coli ST131 infection in patients in a large teaching hospital in China (5). These isolates belonged to serotype O25, as described previously (5). Strains were grown on Columbia blood agar prior to sequencing. The genomic DNA samples were extracted from these three isolates using a DNeasy blood and tissue kit (Qiagen, Germany), according to the manufacturer’s protocols, sequenced on an Illumina HiSeq 4000 sequencer using paired-end 2 × 150-bp reads with approximately 200× coverage, and assembled *de novo* using MicrobeTraker Plus version 0.9.1, with default settings. Read quality was examined using FastQC version 0.11.5. The total numbers of reads of three isolates (EC191, EC192, and EC193) are 5,575,934 bp, 609,608 bp, and 6,033,888 bp, respectively. The read length is 150 bp. The number of contigs and *N*_50_ size of the three E. coli strains (EC191, EC192, and EC193) are as follows: 41 and 343,410 bp, 81 and 401,068 bp, and 111 and 159,190 bp, respectively. The genomes were annotated using NCBI’s Prokaryotic Genome Annotation Pipeline version 4.2 (6). The accession numbers and assembly metrics are listed in Table 1.

**TABLE 1:** Accession numbers and assembly metrics of three draft whole-genome sequences for *E. coli* ST131 O25 strains

Isolate	GenBank accession no.	Genome size (bp)	G+C content (%)	SRA accession no.
EC191	PCFN00000000	5,043,855	50.7	SRR8203406
EC192	PCFO00000000	5,057,410	50.6	SRR8203407
EC193	PCFP00000000	5,266,412	50.7	SRR8203405

### Data availability.

The complete genome sequences of the three E. coli ST131 isolates reported here have been deposited in GenBank under the accession numbers PCFN00000000, PCFO00000000, and PCFP00000000. The BioProject number is PRJNA407669.
